# A36 SERRATED POLYPOSIS SYNDROME OUTCOMES IN THE COLD SNARE RESECTION ERA

**DOI:** 10.1093/jcag/gwae059.036

**Published:** 2025-02-10

**Authors:** A Zarrin, S X Jiang, E Taylor, P Tavakoli, S Bell, A Walia, S Pang, M Yu, D Motomura, R Enns, J Telford, W Xiong, N Shahidi

**Affiliations:** Internal Medicine, The University of British Columbia Faculty of Medicine, Vancouver, BC, Canada; The University of British Columbia Faculty of Medicine, Vancouver, BC, Canada; Stpaul’s Hospital, Division of Gastroenterology, Vancouver, BC, Canada; Stpaul’s Hospital, Division of Gastroenterology, Vancouver, BC, Canada; Internal Medicine, The University of British Columbia Faculty of Medicine, Vancouver, BC, Canada; Internal Medicine, The University of British Columbia Faculty of Medicine, Vancouver, BC, Canada; Stpaul’s Hospital, Division of Gastroenterology, Vancouver, BC, Canada; Internal Medicine, The University of British Columbia Faculty of Medicine, Vancouver, BC, Canada; Gastroenterology, Vancouver Coastal Health Authority, Vancouver, BC, Canada; Stpaul’s Hospital, Division of Gastroenterology, Vancouver, BC, Canada; Stpaul’s Hospital, Division of Gastroenterology, Vancouver, BC, Canada; Stpaul’s Hospital, Division of Gastroenterology, Vancouver, BC, Canada; Stpaul’s Hospital, Division of Gastroenterology, Vancouver, BC, Canada

## Abstract

**Background:**

Surgery is indicated in patients with serrated polyposis syndrome [SPS] when the polyp burden is no longer manageable endoscopically. Evidence has emerged supporting the efficacy and safety of cold snare resection [CSR] for large sessile serrated lesions; however, the role of a primary cold snare resection algorithm [PCSRA] in patients with SPS is unknown

**Aims:**

We aimed to evaluate the efficacy of a PCSRA in patients with SPS

**Methods:**

Utilizing an established retrospective cohort (2013-2023), all patients with a diagnosis of SPS were evaluated, based on the 2019 World Health Organization [WHO] criteria. Patients were stratified by index colonoscopy into the pre-PCSRA (01/2013-12/2020) and the post-PCSRA (01/2021-08/2023). Outcomes included achieving clearance (complete removal of all serrated class lesions [SCL] at procedure completion), adverse events, and referral for surgery. Continuous and categorical variables were summarised using median (IQR) and frequencies respectively. Fisher Exact test was used to compare categorical variables.

**Results:**

178 patients with SPS were included in the analysis (N=144, 80.9% pre-PCSRA; N=34, 19.1% post-PCSRA). Median age at first colonoscopy was 62 years (IQR 9 years) and 115 (64.6%) were female. In the post-PCSRA, 593 SCLs were identified: 326 diminutive (< 5mm), 147 small (5-9mm), 66 medium (10-19mm), and 54 large (≥ 20mm). Among both SCLs and adenomatous lesions, 93.4% (N=627) were removed by CSR. Endoscopic clearance was achieved in 30 (88.2%) patients and 1 patient (2.9%) underwent surgery, which preceded referral for endoscopic management. In comparing the pre-PCSRA vs. the post-PCSRA: 110 (76.4%) achieved clearance in the pre-PCSRA group (P=0.09). No delayed bleeding was observed in the post-PCSRA group as opposed to 3 (2.2%) in pre-UCSRA (p = 0.50). Intra-procedural perforation was reported in 1 patient in the post-PCSRA vs. 2 in the pre-PCSRA group (p=0.47). Only 1 delayed perforation was reported in the pre-PCSRA group (p=0.81). Twenty-one (13.9%) patients underwent surgery: 20 in the pre-PCSRA (94.7%; 9 due to polyp burden and 11 for colorectal cancer) and 1 in the post-PCSRA (colorectal cancer) (p=0.08).

**Conclusions:**

Cold snare resection should be considered as the first-line resection modality for most lesions identified in patients with SPS.

**Funding Agencies:**

None

